# Protegrin-1 Combats Multidrug-Resistant Porcine ExPEC: Potent Bactericidal Activity and Multimodal Immunometabolic Regulation In Vitro and in a Murine Model

**DOI:** 10.3390/vetsci12111030

**Published:** 2025-10-23

**Authors:** Jing Xu, Yinlin He, Zihao Liang, Shengfeng Chen, Biao Tang, Fei Su, Canying Liu

**Affiliations:** 1 College of Animal Science and Technology, Foshan University, Foshan 528225, China; 2 Key Laboratory of Systems Health Science of Zhejiang Province, School of Life Science, Hangzhou Institute for Advanced Study, University of Chinese Academy of Sciences, Hangzhou 310024, China; 3 Institute of Animal Husbandry and Veterinary Medicine, Zhejiang Academy of Agricultural Sciences, Hangzhou 310021, China

**Keywords:** protegrin-1, spleen, inflammation, extraintestinal pathogenic *Escherichia coli*, transcriptome

## Abstract

**Simple Summary:**

As antibiotics become less effective against certain bacteria, finding new treatments is increasingly important. This study focused on a drug-resistant strain of *E. coli* that causes severe infections in pigs and poses a threat to humans. We investigated a natural substance called Protegrin-1 (PG-1) as a potential solution. Our experiments showed that PG-1 successfully killed the drug-resistant bacteria in the lab, and the bacteria did not develop resistance to it. In mice with infections, PG-1 treatment greatly increased survival rates, cleared bacteria from major organs, and repaired tissue damage. A key finding was that PG-1 works in two ways: it directly kills the bacteria while also helping to control the body’s own damaging inflammatory response. This dual action makes PG-1 a very promising candidate for developing new therapies against infections that are resistant to conventional antibiotics.

**Abstract:**

Porcine extraintestinal pathogenic *Escherichia coli* (ExPEC) is a significant zoonotic pathogen with escalating antimicrobial resistance, underscoring the urgent need for novel therapeutics. This study aimed to investigate the therapeutic potential and mechanism of action of the antimicrobial peptide Protegrin-1 (PG-1) against a multidrug-resistant porcine ExPEC strain, PCN033. The minimal inhibitory concentration (MIC) was determined, and resistance stability was assessed through serial induction and withdrawal passages. Hemolytic activity was evaluated to gauge selectivity. A murine infection model was utilized to assess in vivo efficacy, bacterial load reduction, cytokine modulation, and histopathology. Comparative spleen transcriptomic analysis was performed to elucidate global host responses. PG-1 exhibited potent bactericidal activity (MIC = 32 μg/mL) and maintained its efficacy over multiple passages, demonstrating no induced resistance. It showed acceptable hemolytic activity and significantly improved survival, reduced bacterial loads in multiple organs, and mitigated tissue damage in mice. Transcriptomics revealed PG-1 treatment broadly tempered infection-induced hyperinflammatory responses, including NF-κB, MAPK, and TNF signaling pathways, and counteracted metabolic reprogramming. The findings conclude that PG-1 effectively integrates direct, resistance-resistant bactericidal activity with multimodal immunomodulation, representing a superior therapeutic strategy that simultaneously eliminates pathogens and restores immune homeostasis, offering a promising alternative to conventional antibiotics against MDR ExPEC infections.

## 1. Introduction

The escalating global antimicrobial resistance (AMR) crisis poses a formidable threat to both human and animal health, undermining the efficacy of conventional antibiotics and compromising modern medical advances [1]. This challenge is particularly acute in the case of extraintestinal pathogenic *Escherichia coli* (ExPEC) [2]. While the majority of *E. coli* strains exist as harmless commensals within the intestinal tract, ExPEC variants have evolved sophisticated mechanisms to colonize extraintestinal sites and cause severe infections. The clinical manifestations of ExPEC-mediated diseases include urinary tract infections (UTIs), neonatal meningitis, surgical site infections, and sepsis, each contributing to a substantial global healthcare burden [3]. The pathogenicity of ExPEC is defined by a multi-step process mediated by diverse virulence factors: (1) colonization of mucosal surfaces through specific adhesins; (2) invasion and survival within host cells; (3) nutrient acquisition under iron-limited conditions; and (4) evasion of host immune responses, often mediated by capsule and lipopolysaccharide. The synergy of these mechanisms facilitates colonization, immune evasion, and host damage [4,5,6]. Compounding this public health crisis is the emergence and global dissemination of porcine ExPEC strains. These pathogens cause severe systemic infections in swine, characterized by polyserositis and meningitis, resulting in significant economic losses due to increased mortality, reduced growth rates, and rising veterinary costs. Simultaneously, porcine ExPEC strains act as a reservoir of highly diverse and mobile antimicrobial resistance genes, including those conferring resistance to critically important human antibiotics such as extended-spectrum cephalosporins, fluoroquinolones, colistin, and carbapenems. Furthermore, the frequent sharing of virulence gene profiles and sequence types between porcine and human ExPEC isolates underscores a serious zoonotic threat [7,8,9,10,11]. This convergence of factors severely limits treatment options and intensifies the AMR crisis, highlighting the urgent need for novel antimicrobial strategies.

Antimicrobial peptides (AMPs) have emerged as promising alternatives to conventional antibiotics due to their unique mechanism of action and lower propensity for resistance development. As key components of innate immunity across diverse species, AMPs primarily target and disrupt microbial membranes, a fundamental and conserved cellular structure that bacteria cannot easily alter without compromising viability. This mode of action contrasts with that of traditional antibiotics, which inhibit specific enzymes or processes and are thus more susceptible to target-based resistance mutations. Moreover, many AMPs exhibit broad-spectrum activity and dual functionality, capable of both killing pathogens and modulating host immune responses. However, their clinical translation faces challenges, including potential cytotoxicity (e.g., hemolysis), susceptibility to proteolytic degradation, and limited bioavailability. Therefore, a comprehensive evaluation of their efficacy, safety, and stability is essential for advancing viable AMP candidates [12,13,14]. Among AMPs, protegrin-1 (PG-1), an 18-amino acid cathelicidin derived from porcine leukocytes, demonstrates exceptional therapeutic potential. It exhibits potent antibacterial and antiviral activities, coupled with immunomodulatory properties. The antimicrobial mechanism of PG-1 involves electrostatic attraction to anionic bacterial membranes, followed by integration into the lipid bilayer and subsequent transmembrane pore formation, leading to uncontrolled ion flux, collapse of membrane potential, and ultimately rapid bacterial cell death. Beyond direct microbial killing, PG-1 neutralizes lipopolysaccharide (LPS) endotoxin by binding to lipid A and preventing its recognition by host pattern recognition receptors, thereby attenuating septic shock cascades [15,16]. Emerging evidence highlights the critical role of PG-1 in immune regulation. It fine-tunes inflammatory responses to enhance microbial clearance while minimizing tissue damage. At the molecular level, PG-1 interacts with various inflammatory triggers, including LPS, capsular polysaccharides and other pathogen-associated molecular patterns, suppressing excessive innate immune activation, particularly in macrophages. In experimental models, PG-1 inhibits macrophage hyperactivation and normalizes pro-inflammatory cytokines such as IL-1β, IL-6, and TNF-α, mitigating uncontrolled inflammation without compromising host defense [17,18,19,20,21]. The peptide also significantly promotes tissue repair across multiple physiological contexts. In a *Staphylococcus aureus*-infected wound model, PG-1-overexpressing transgenic mice exhibited complete infection resolution and accelerated neo-epithelium formation without antibiotic intervention [22]. This reparative function is further elaborated in intestinal models, where PG-1 administration in *Citrobacter rodentium*-infected mice promoted mucosal healing through coordinated upregulation of repair genes (TFF3 and MUC-2) and normalization of inflammatory mediators (IL-1β, IL-6). PG-1 simultaneously accelerated intestinal epithelial cell migration via IGF1R activation, a mechanism conserved in dermal keratinocytes, while modulating immune signaling through pathways involving CCL2 and NF-κB. This multifaceted capacity, observed in diverse cell types including porcine ovarian granulosa cells, confirms the broad tissue tropism of PG-1 and positions it as a multifunctional therapeutic candidate that orchestrates both tissue repair and immunomodulation [16,20,23,24,25,26,27]. Despite these advanced capabilities, the precise mechanisms underlying immunomodulatory actions of PG-1, particularly in complex MDR ExPEC infections and their impact on host metabolic responses, remain poorly understood.

To address these critical knowledge gaps, this study aims to comprehensively evaluate the therapeutic potential of PG-1 against MDR porcine ExPEC using an integrated multidisciplinary approach. Our specific objectives include: (1) determining the in vitro bactericidal activity and resistance stability of PG-1 against the MDR-virulent porcine ExPEC strain PCN033; (2) assessing its potential toxicity via hemolytic activity assays; (3) evaluating its in vivo protective efficacy in a murine systemic infection model by monitoring survival, bacterial burden, cytokine profiles, and histopathological changes; and (4) elucidating the molecular mechanisms of the action of PG-1 through comparative spleen transcriptomic analysis against tetracycline as a conventional antibiotic control, with emphasis on its immunomodulatory and metabolic regulatory functions. This research will provide crucial insights for developing PG-1-based therapeutic strategies against challenging MDR bacterial infections, potentially paving the way for a new class of anti-infectives that simultaneously target pathogens and modulate host responses for improved clinical outcomes.

## 2. Materials and Methods

### 2.1. Bacterial Strains

ExPEC PCN033 [GenBank: CP006632.1] is a multidrug-resistant strain isolated from the brain of a diseased swine in China. Additionally, PCN033 presented as a highly virulent ExPEC strain in the mouse and pig model [8,10]. *E. coli* ATCC25922 [GenBank: OP984756.1] was preserved by the Veterinary Medicine Laboratory at Foshan University and used as quality control. Both strains were cultured in Luria–Bertani (LB) broth or on LB agar plates at 37 °C.

### 2.2. In Vitro Antibacterial Activity Assay

The antibacterial activities of synthesized mature PG-1 (Shanghai Top-Peptide Biotechnology Co., Ltd., Shanghai, China, the synthesized amino acid sequence of mPG-1: RGGRLCYCRRRFCVCVGR, purity ≥ 95%) and tetracycline (Gen-View Scientific Inc., Beijing China, product No.: AT341-10G) against PCN033 and *E. coli* ATCC25922 were evaluated by the minimum inhibitory concentration (MIC) measurement via broth microdilution method [28]. The PG-1 and tetracycline was dissolved in LB and diluted at concentrations of (128—0.25 μg/mL) and (1024—8 μg/mL) by twofold dilutions in a 96-well plate, respectively. Afterwards, 50 µL of serial dilutions were inoculated with an equal volume of logarithmic phase bacterial suspensions (1 × 10^8^ CFU/mL) and incubated at 37 °C for 16–20 h. The MIC was defined as the lowest concentration of a compound to completely inhibit visible bacterial growth according to the guidelines (M27-A3) (CLSI 2008). Minimum bactericidal concentration (MBC) was determined by plating 50 µL of medium from non-turbid wells onto LB agar [28]. After overnight incubation at 37 °C, viable colonies were counted. The tests used sterile LB broth as negative control. Each treatment was repeated three times.

### 2.3. In Vitro Synergistic Activity of PG-1 and Tetracycline

The synergistic antibacterial activity of PG-1 combined with tetracycline against PCN033, and the fractional inhibitory concentration (FIC) indices were measured by checkerboard assays, Briefly, 25 µL of twofold serial dilutions of PG-1 ranging from 8 MIC to 1/16 × MIC were added along the abscissa while 25 µL of twofold serial dilutions of tetracycline ranging from 8 × MIC to 1/16 × MIC were added along the ordinate in 96-well plates. Then, 50 µL of logarithmic phase bacterial suspensions (1 × 10^8^ CFU/mL) were added. Next, the 96-well plates were incubated at 37 °C for 18 h and examined for visible turbidity. The FIC index was calculated to evaluate the interactive profile of the combination of AMP PG-1 and antibiotic tetracycline, using the following formula:

FIC index = (MIC of AMP in combination/MIC of AMP alone) + (MIC of antibiotic in combination/MIC of antibiotic alone).

The FIC index values were defined as follows: FIC < 0.5 indicates synergistic effects, 0.5 < FIC ≤ 0.75 indicates partial synergy effects, 0.75 < FIC ≤ 1 indicates additive effect, FIC > 1 indicates indifferent effect, FIC > 4 indicates antagonistic effect [29].

### 2.4. Antimicrobial Resistance Induction In Vitro

Antimicrobial resistance induction of ExPEC PCN033 strain was performed as previously reported with modification [30]. A logarithmic phase bacterial suspension (1 × 10^8^ CFU/mL) underwent 30 generations of serial passage in LB broth with escalating antimicrobial concentrations: 1/4 × MIC (generations 1–10), 1/2 × MIC (generations 11–20), and MIC (generations 21–30) of tetracycline (TET) or PG-1 under standard conditions (37 °C, 120 rpm, 24 h/passage). After induction, cultures were transferred to drug-free LB broth for 10 additional passages to monitor resistance persistence. Bacterial stocks were cryopreserved in 20% glycerol at −80 °C every 3 generations. MIC values for both antimicrobials were determined every third generation using broth microdilution method according to CLSI guidelines (M07-A11). The change in MIC was described by normalizing the MIC of the n generation to the MIC of first generation.

### 2.5. Haemolytic Activity Assay

The haemolytic activity of PG-1 was assessed using mouse erythrocytes via a modified version of the previously described method [31]. Blood samples (1 mL) were collected from BALB/c mice to prepare a 10% (*w*/*w*) erythrocyte suspension. Aliquots of 200 µL of the erythrocyte suspension were mixed with an equal volume of PG-1 solution and incubated at 37 °C for 1 h. The PG-1 solution was evaluated within the concentration range of 1/32 × MIC to 4 × MIC. PBS and 0.1% Triton-X-100 (Sigma-Aldrich, St. Louis, MO, USA) served as the negative control and positive control, respectively. After incubation, the mixtures were centrifuged at 1200× *g* for 5 min at 4 °C. Subsequently, 200 µL of the supernatant from each sample was transferred to a 96-well plate, and the absorbance at 540 nm (OD_540_ nm) was measured using a microplate reader (iMark™ Microplate Absorbance Reader, Bio-Rad Laboratories, Inc., Hercules, CA, USA, Model #1681130). The hemolysis rate was calculated using the following formula:

Hemolysis rate (%) = (OD_540_ nm of treated sample − OD_540_ nm of negative control)/(OD_540_ nm of positive control − OD_540_ nm of negative control) × 100%

### 2.6. In Vivo Efficacy of PG-1 in Mouse PCN033 Infection Model

All mouse experiment procedures were performed adhered to protocols approved by the Institutional Animal Care and Use Committee (IACUC) of Foshan University (Approval No.: FOSU190901). Four-week-old female BALB/c mice were purchased from Guangdong (China) and housed under a specific pathogen-free environment, following a 12 h light/dark cycle, at a controlled temperature (22 ± 2 °C). Mice were intraperitoneally challenged with 1 × 10^7^ CFU of PCN033 in 200 µL sterile PBS. The control mice were injected with the same volume of PBS (Control group). The infected mice were randomly assigned to 3 groups as follows: PCN033-infected mice without treatment receiving 200 µL sterile PBS (PBS group), tetracycline group (TET group) and PG-1 group were intraperitoneally administered 30 mg/kg of tetracycline and 3.2 mg/kg PG-1 1 h post-infection, respectively. Afterwards, survival rates, bacterial load quantification, histopathological analysis, and cytokine level measurements were evaluated. Survival of the mice (*n* = 16 per group) was monitored every 6 h for 72 h post treatment with observations recorded at each interval. At 12 h post-infection, mice (*n* = 5 per group) were euthanized under anesthesia using ether. Then, organs (including spleen, brain, and lung) and blood were aseptically collected, homogenized, and plated on LB agar for bacterial load quantification via serial tenfold dilution. After overnight incubation at 37 °C, the number of bacterial colonies was counted and expressed as CFU/g of organ or CFU/mL of blood. Histopathological examination (mice, *n* = 3 per group) was performed 12 h post-infection. Tissue samples from spleen, lungs, and brains, were fixed in 10% formalin for more than a week, embedded in paraffin, and sectioned at 4–5 µm thickness for hematoxylin and eosin staining [32]. For cytokine detection, mice (*n* = 3 per group) were euthanized 12 h post-infection. Tissue homogenates from lung, spleen, brain along with serum samples were analyzed for IL-1β, TNF-β, and TGF-α levels using ELISA kits (Jiangsu Enzyme Immunoassay Industrial Co., Ltd., Yancheng, China, IL-1β: Cat. No. MM-0905M1, TNF-β: Cat. No. MM-0131M1, and TGF-α: Cat. No. MM-0122M1) according to the manufacturer’s protocols.

### 2.7. RNA Isolation

Total RNA was extracted from spleens of mice in the control group, PBS group, PG-1 group, and TET group (*n* = 3 per group) using TRIzol reagent (Invitrogen, Carlsbad, CA, USA). Spleen tissue samples were placed into RNase-free EP tubes and homogenized in RLTPlus lysis buffer (Kangwei Century (Beijing, China), Cat. No. CW0599S) using a cryogenic tissue homogenizer (TissueLyserⅡ, Qiagen, Hilden, Germany). Total RNA was isolated from the homogenized tissue samples by the tissue RNA extraction kit (Kangwei Century, Cat. No. CW0599S) following the manufacturer’s instructions. For each sample, 2 μg of total spleen RNA was treated with DNase I (Sigma-Aldrich, St. Louis, MO, USA) to eliminate genomic DNA contamination. Then, the RNeasy Mini Kit (Qiagen, Dusseldorf, Germany) was used to purify the RNA. RNA integrity and concentration quantification were assessed using the Bioanalyzer 2100 system (Agilent Technologies, Santa Clara, CA, USA). RNA samples were flash-frozen in liquid nitrogen and stored at −80 °C until further processing.

### 2.8. Transcriptome Analysis

The RNA samples were delivered to Novogene Bioinformatics Technology Co., Ltd. (Beijing, China) for cDNA library construction and paired-end sequencing on an Illumina NovaSeq 6000 platform (Illumina, Inc., San Diego, CA, USA). Raw reads in FASTQ format were initially processed using the fastp (v0.23.2) software to obtain clean data. Processed clean reads were evaluated for sequencing quality using Q20/Q30 scores and GC content distribution. Paired-end clean reads with high quality were aligned to the BALB/c mouse reference genome (Mus musculus, GCA_001632525.1) using Hisat2 v2.0.5. The mapped reads of each sample were assembled by StringTie (v1.3.3b) [33] in a reference-based approach. Read counts for each gene were obtained using FeatureCounts v1.5.0-p3, and the fragments per kilobase of transcript per million mapped reads (FPKM) were calculated for each gene. Differentially expressed genes (DEGs) were identified using the DESeq2 (v1.20.0) in R (v4.2.1) with significance thresholds set at adjusted *p*-value (Benjamini–Hochberg) < 0.05 and |log_2_(fold change)| > 1. Gene Ontology (GO) and Kyoto Encyclopedia of Genes and Genomes (KEGG) enrichment analyses of the DEGs were performed using clusterProfiler (v3.8.1). Statistical significance for enrichment results was determined using Fisher’s exact test with false discovery rate (FDR) correction (adjusted *p* < 0.05).

### 2.9. Eukaryotic Reference-Based Transcriptome Validation Using RT-qPCR

Total RNA extracted from spleen tissue was reverse-transcribed into first-strand cDNA using the PrimeScript™ RT Reagent Kit (Takara Biotechnology, Dalian, China) in a Biometra TProfessional Standard 96 Gradient thermocycler (Biometra, Göttingen, Germany). Quantitative real-time PCR was conducted to amplify cDNA using 2 × SYBR Green qPCR Master Mix (Servicebio, Wuhan, China) and performed on LightCycler^®^ 96 Real-Time PCR System (Roche Diagnostics, Basel, Switzerland). Two housekeeping genes glyceraldehyde-3-phosphate dehydrogenase (*GAPDH*) and *β-actin* were used as internal controls for data normalization. All reactions were conducted in triplicate using the following thermal cycling parameters: initial denaturation at 95 °C for 3 min, followed by 40 cycles of 95 °C for 10 s and 60 °C for 30 s. Melt curve analysis was performed to verify reaction specificity. Relative gene expression levels were calculated using the comparative 2^−ΔΔCt^ method, with normalization against both reference genes [34]. The changes in gene expression were reported as fold-increases relative to controls. Primer sequences used in this study are detailed in Table 1.

### 2.10. Control Groups and Procedures

Appropriate controls were included in all experiments to ensure reliability and validity. For in vitro antibacterial assays, sterile culture broth served as the negative control. In the hemolytic activity assay, PBS and 0.1% Triton-X-100 were used as negative and positive controls, respectively. For the in vivo mouse model, a group of uninfected mice injected with PBS was included as the healthy control (Control group). Infected mice treated with PBS served as the disease model control (PBS group). In all molecular biology procedures, including RNA extraction, reverse transcription, and qPCR, no-template controls (NTCs) were included to monitor for contamination.

### 2.11. Statistical Analysis

Statistical analysis was conducted using GraphPad Prism 9.0 software (San Diego, CA, USA). All data are presented as means ± standard error of the mean (SEM). For comparisons between two groups, Student’s *t*-test was used. For comparisons among multiple groups, one-way analysis of variance (ANOVA) followed by Tukey’s multiple comparison test was performed. The value of *p* < 0.05 was considered statistically significant.

## 3. Results

### 3.1. Antibacterial Activity of PG-1 and Tetracycline

The antibacterial efficacy of PG-1 and TET was quantitatively evaluated using two parameters: the MIC and MBC. As detailed in Table 2, PG-1 exhibited identical MIC values of 32 μg/mL against both ExPEC PCN033 and *E. coli* ATCC25922 strains, demonstrating bacterial activity at 32 μg/mL for PCN033 and 64 μg/mL for ATCC25922. In contrast, TET showed limited antibacterial activity against PCN033, with MIC and MBC at 512 μg/mL, while displayed potent inhibition of *E. coli* ATCC25922 with MIC at 0.125 μg/mL and MBC at 0.25 μg/mL. Detailed results are presented in Table 2.

### 3.2. Synergistic Antibacterial Effect of PG-1 in Combination with TET In Vitro

A checkerboard assay was performed to characterize the antibactericidal interaction between the AMP PG-1 and antibiotic TET against PCN033. As presented in Table 3, the combinatorial treatment revealed a FIC index of 1.03125, which corresponds to an indifferent antibactericidal effect based on established criteria (FIC > 1.0 defines indifference interaction).

### 3.3. Induction of Antibiotic Resistance In Vitro

**Figure 1:**
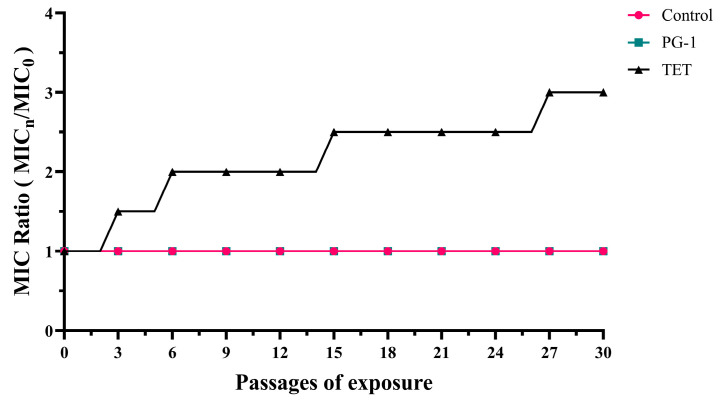
The effect of PG-1 or TET on drug resistance in PCN033. PCN033 was cultured escalating antimicrobial concentrations: 1/4 × MIC (generations 1–10), 1/2 × MIC (generations 11–20), and MIC (generations 21–30) of tetracycline (TET) or PG-1 under standard conditions (37 °C, 120 rpm, 24 h/passage). MIC changes were determined by normalizing the MIC of the n generation to that of the first generation.

The development of bacterial resistance is frequently associated with inappropriate antibiotics usage. To evaluate the resistance-inducing potential of AMP PG-1 versus antibiotic TET, we performed a serial passaging experiment with PCN033 using stepwise antibiotic escalation from 1/4 × MIC to 1 × MIC over 30 generations. As demonstrated in Figure 1, TET exposure induced progressive resistance development, with serial passage under antibiotic pressure demonstrating stepwise MIC elevation: 0.5-fold increase at passage 3, escalating to 1-fold, 1.5-fold, and ultimately 2.5-fold at passages 6, 15, and 27, respectively, revealing a positive correlation between exposure duration and resistance level. Conversely, PG-1 exhibited remarkable antimicrobial stability, maintained MIC values (1 × MIC) throughout both the initial 30-passage induction phase and an extended 10-generation withdrawal period (generations 31–40) in PG-1-free medium (Table S1).

### 3.4. Hemolytic Analysis

**Figure 2:**
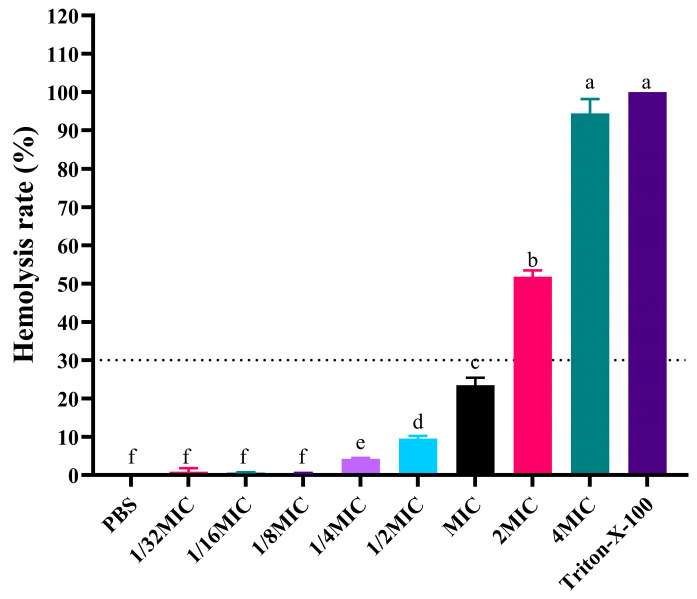
Hemolytic activity of PG-1 in mouse red blood cells. PBS and 0.1% Triton-X-100 served as the negative control and positive control, respectively. The dashed line indicates the 30% hemolysis threshold, a benchmark for acceptable cytotoxicity. The same lowercase letters indicated no significant difference (*p* > 0.05), while different lowercase letters denote significant differences (*p* < 0.05).

As shown in Figure 2, PG-1 exhibited concentration-dependent hemolytic activity with controlled cytotoxicity across the therapeutic concentration ranging from 1/32 × MIC (1 μg/mL) to MIC (32 μg/mL), during which the hemolysis rate was maintaining below 23.47 ± 1.91%. Notably, cytotoxic threshold emerged at 2 × MIC (64 μg/mL), inducing 51.82 ±1.66% hemolysis. At 4 × MIC (128 μg/mL), near-complete erythrocyte lysis (94.40 ± 3.78%) occurred (Table S1).

### 3.5. Anti-Infective Effects of PG-1 and Tetracycline Against PCN033 Infection In Vivo

**Figure 3:**
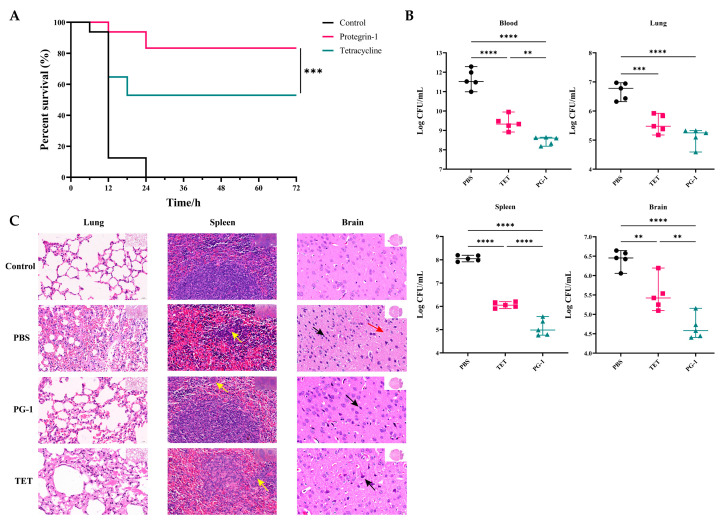
Therapeutic effects of PG-1 or TET on mouse infection with ExPEC PCN033. Mice were intraperitoneally challenged with 1 × 10^7^ CFU of PCN033 and then intraperitoneally administered with PG-1 or TET. The infected mouse treated with PBS were designated as the PBS group. (**A**) Survival rate of mice treated with PG-1 or TET (*n* = 16). (**B**) Bacterial load in the blood, lung, spleen, and brain of PCN033-infected mice was determined (*n* = 5). (**C**) H&E staining of lung, spleen, and brain tissues were performed at 12 h post-infection for histological analysis (original magnification ×400). Yellow arrows indicate macrophage. Black arrows indicate degenerated neurons. Red arrows indicate interstitial space in some areas of the cerebral cortex is enlarged. Control: normal group; PBS: model group; PG-1: 3.2 mg/kg PG-1 treatment group; TET: 30 mg/kg TET treatment group (*n* = 3). Images are representative of those of three mice from each group. Statistical significance is indicated by ** *p* < 0.01, *** *p* < 0.001, and **** *p* < 0.0001.

**Figure 4:**
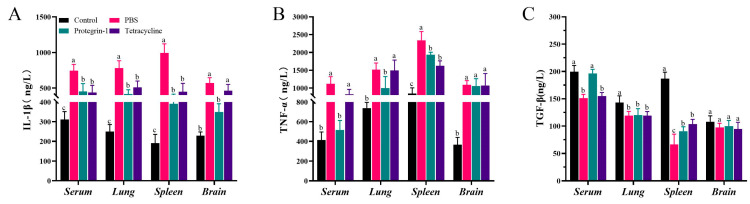
The effects of PG-1 or tetracycline on the production of inflammatory cytokines. Mice were intraperitoneally infected with PCN033 (1 ×  10^7^ CFU) and then treated with tetracycline or PG-1. The infected mice were treated with PBS served as an infection model. At 12 h post-infection, the production of inflammatory cytokines, including IL-1β (**A**), TNF-α (**B**), TGF-β (**C**) in the serum, lung, spleen, and brain was determined using ELISAs (*n* = 3/group). The same lowercase letters indicated no significant difference (*p* > 0.05), while different lowercase letters denote significant differences (*p* < 0.05).

To evaluate the anti-infective efficacy of PG-1 and tetracycline in vivo, we established a mouse infection model challenged with PCN033. Survival analysis (Figure 3A) shown that all mice in the PBS group dead within 24 h post-infection. PG-1 treatment resulted in an 87.5% survival rate (14/16) within 24 h, whereas TET-treated mice exhibited an 50% (8/16) survival rate within 18 h (Table S1). Additionally, PG-1 and TET treatment both significantly reduced the bacterial loads in the blood, brain, spleen and lung of infected mice, while PG-1 demonstrated significantly greater bacterial clearance efficacy compared with TET in the blood, brain, and spleen of infected mice (Figure 3B) (Table S1). As shown in Figure 3C, H&E staining of pathological sections revealed significant pulmonary changes. In the lung tissue, the PBS group exhibited significant damage, including extensive alveolar atrophy and collapse, epithelial cell hyperplasia, thickened alveolar walls, and inflammatory cell infiltration. In the TET group, the lung damage improved but some alveolar shrinkage and inflammation remained. In the PG-1 group, the lung injury was further reduced, with more normal alveoli and minimal inflammation. In the spleen tissue, the PBS group showed disordered splenic nodules and reduced lymphocytes, while the TET group had some relief of damage and the PG-1 group had essentially normal spleen tissue with sufficient lymphocytes. The brain tissue results indicated obvious neuron degeneration in the PBS group, fewer degenerative neurons in the TET group, and further reduction of neuron degeneration in the PG-1 group. Overall, both PG-1 and TET ameliorated tissue damage, but PG-1 was more effective, showing better tissue protection and repair. To further evaluate the effects of PG-1 and TET on systemic inflammation following PCN033 infection, levels of pro-inflammatory cytokines (IL-1β and TNF-α) and anti-inflammatory cytokine (TGF-β) were measured in the serum, lungs, spleens and brains of mice. As shown in Figure 4, PCN033 infection elevated IL-1β and TNF-α levels in serum, lungs, spleens and brains while reducing TGF-β in serum, lungs, and spleens. PG-1 treatment suppressed IL-1β levels in all examined tissues, inhibited TNF-α in serum, lungs, and spleens, and elevated TGF-β levels in serum and spleens, demonstrating significant immunomodulatory properties. In contrast, TET reduced IL-1β levels in serum, lungs, and spleens, decreased TNF-α levels specifically in the spleens, and increased TGF-β in the spleens (Table S1). Collectively, these results indicate that PG-1 possesses superior potent antibacterial activity and enhanced immunomodulatory capacity compared to TET, effectively mitigating infection-induced systemic inflammation.

### 3.6. Transcriptome Sequencing and Read Mapping

Spleen samples were collected from mice of four groups: the control, PBS, PG-1, and TET group (*n* = 3 per group). Transcriptome sequencing of these twelve samples generated 556,985,936 raw reads. Following quality control and data filtering, each sequencing library generated more than 39,509,588 clean reads, with Q30 exceeding 97.19%, and the G/C content was approximately 49.77%. The average percentage of sequences mapped onto the murine reference genome was 89.05%, resulted in 483,704,890 mapped reads (Table 4). Raw sequencing data were deposited in the NCBI Sequence Read Archive (SRA) under Bioproject accession number PRJNA1280477.

### 3.7. Analysis of Differentially Expressed Genes (DEGs)

**Figure 5:**
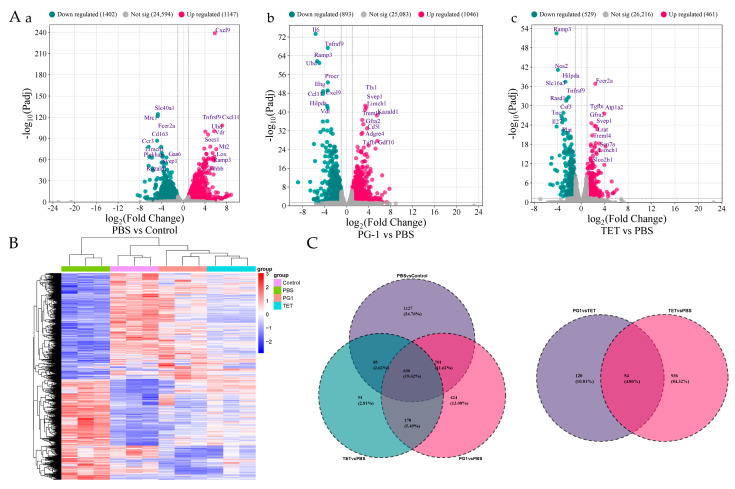
Transcriptome profile comparison between groups. (**A**) Volcano plots of differentially expressed genes (DEGs). DEGs were defined by |log_2_ (FoldChange)| > 1 and adjusted *p*-value < 0.05. Significantly upregulated genes are shown in red, downregulated genes in blue, and non-DEGs in gray. (**a**) Effect of PCN033 challenge on gene expression. (**b**) Comparison between PCN033 challenge and PG-1 treatment. (**c**) Comparison between PCN033 challenge and TET treatment. (**B**) Heatmap of the expression patterns of all identified DEGs. Rows represent individual genes, and columns represent biological samples. Expression levels are color-coded, with red indicating upregulated and blue indicating downregulated genes. (**C**) Venn diagram depicting the number and proportion of unique and overlapping DEGs across the different experimental groups. Control: uninfected group; PCN033: PCN033-challenged group; PG-1: PCN033-challenged group treated with PG-1 (3.2 mg/kg); TET: PCN033-challenged group treated with TET (30 mg/kg) (*n* = 3).

To identify DEGs in mouse spleen, pairwise comparisons were conducted between: (1) PBS vs. Control group; (2) PG-1 vs. PBS group; (3) TET vs. PBS group. Volcano plot visualized DEGs distribution across all pairwise group comparisons (Figure 5A). Overall, PCN033 infection (PBS vs. Control) induced 2549 DEGs, including 1147 upregulated and 1402 downregulated genes. PG-1treatment (PG-1 vs. PBS) exhibited 1939 DEGs with 1046 upregulated and 893 downregulated genes, significantly more than TET treatment (TET vs. PBS) showing 990 DEGs among which 461 were upregulated and 529 were downregulated. Comprehensive lists of significantly DEGs for each comparison are provided in Table S2, while Table S3 details the top ten significantly up- and down-regulated DEGs per group. Notably, PG-1 treatment downregulated 6 genes ranking among the top 10 most upregulated genes following PCN033 infection, including C-X-C motif chemokine 9 (*Cxcl9*), C-X-C motif chemokine 11 (*Cxcl11*), Ubiquitin D (*Ubd*), Tumor necrosis factor receptor superfamily member 9 (*Tnfrsf9*), Vitamin D3 receptor (*Vdr*), and Receptor activity-modifying protein 3 (*Ramp3*). Among these, *Ramp3* and *Tnfrsf9* were also downregulated following TET treatment. Notably, *Cxcl9* and *Cxcl11* encode pro-inflammatory mediators, while *Ubd*, *Tnfrsf9*, *Vdr* and *Ramp3* were also linked to inflammatory response [35,36,37,38]. Expression patterns of these DEGs across twelve transcriptome sequencing samples are shown in the heatmap (Figure 5B), genes with similar expression patterns were clustered. Venn diagram (Figure 5C) identified 814 co-expressed DEGs shared across treatment, with PG-1 and TET exhibiting 1125 and 176 unique DEGs versus PBS, respectively.

**Figure 6:**
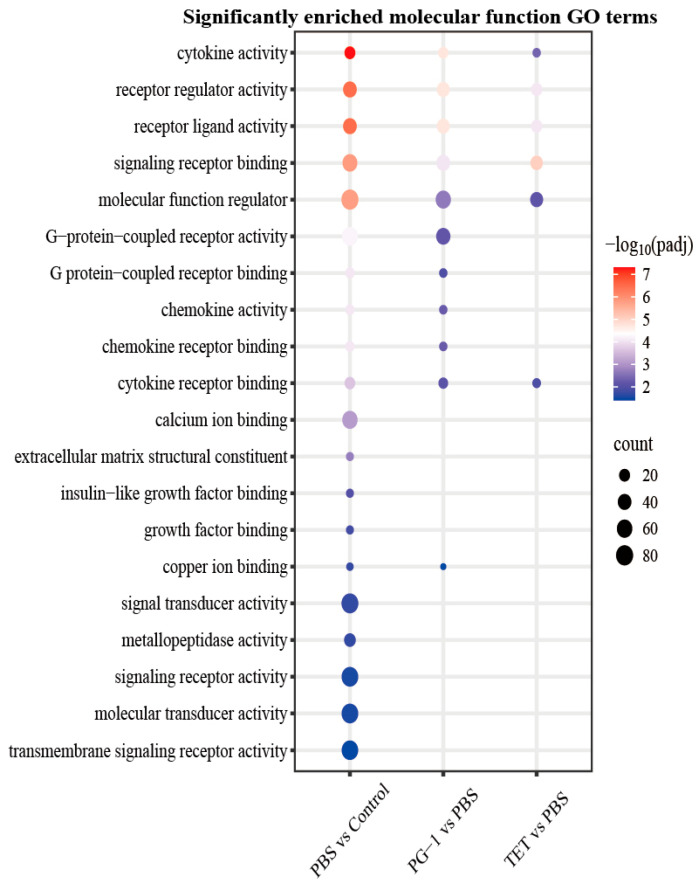
GO enrichment analysis. Dot plots show significantly enriched GO terms of molecular functions under the different comparison groups (adjusted *p*-value < 0.05). The size of the dot is based on gene count enriched in the GO terms, and the color of the dot from red to blue shows the GO terms enrichment significance.

GO analysis characterized the functional roles of these DEGs (Figure 6, Table S4). All comparisons showed significant enrichment for the cellular component “extracellular region” and the biological process “immune system process”. The top five significantly enriched molecular function terms across comparisons were consistent, including cytokine activity, receptor regulator activity, receptor ligand activity, signaling receptor binding and molecular function regulator, though profiles differed. The PG-1 vs. PBS group displayed unique significant enrichment for G-protein-coupled receptor activity, G-protein-coupled receptor binding, chemokine activity, and chemokine receptor binding. Furthermore, DEGs in the PBS vs. Control group were specifically enriched in immune response, organic acid biosynthetic process, carboxylic acid biosynthetic process, and cell adhesion, whereas DEGs in the TET vs. PBS group were predominantly enriched in tetrapyrrole metabolic process.

**Figure 7:**
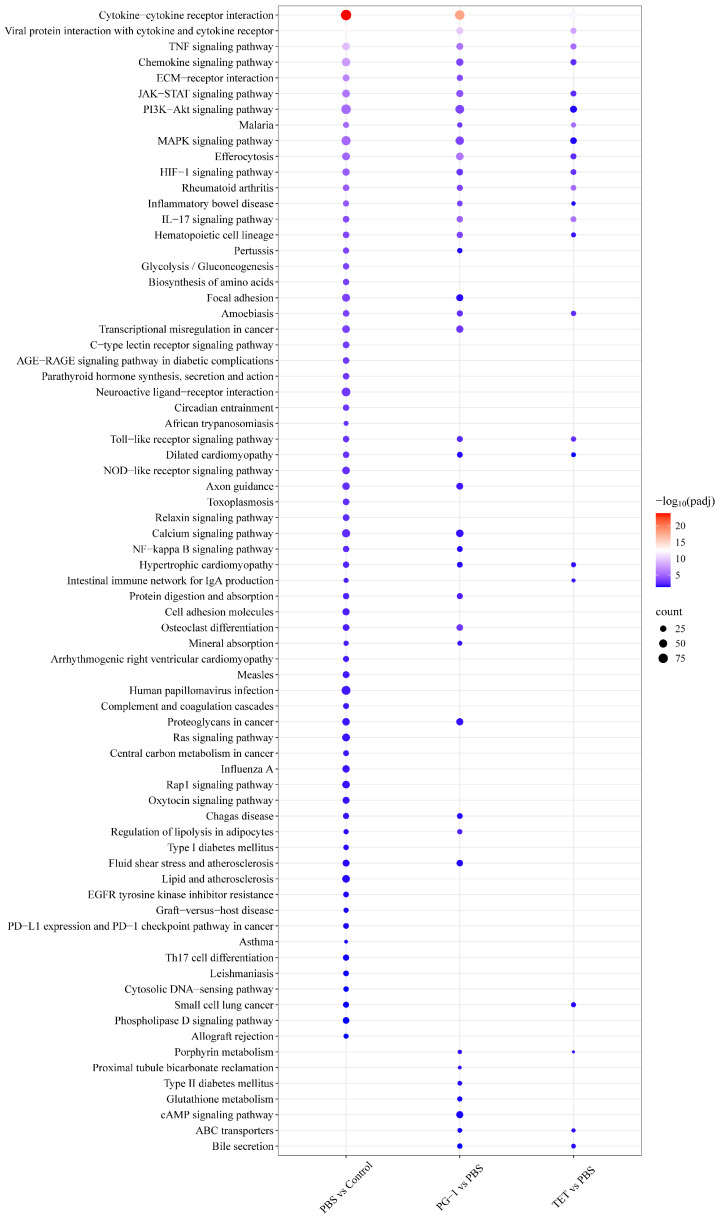
KEGG pathway enrichment analysis. Dot plots show significantly enriched KEGG pathways under the different comparison groups (adjusted *p*-value < 0.05). The size of the dot is based on gene count enriched in the pathway, and the color of the dot from red to blue shows the pathway enrichment significance.

To investigate response pathways in the murine spleen altered by PCN033 infection and PG-1/TET treatment, DEGs were mapped to the KEGG database. KEGG pathways analysis classified enriched pathways into five categories (Table S5, Figure S1) including metabolism, environmental information processing, cellular processes, organismal systems, and human diseases. PBS vs. Control, PG-1 vs. PBS, and TET vs. PBS comparisons yielded 66, 39, and 23 significantly enriched pathways, respectively (Table S5), primarily involving signal transduction, signaling molecules and interaction, and immune systems. Additionally, 18 pathways were shared across all three comparisons (Figure 7, Table S5), predominantly clustered in the categories of signaling molecules and interaction (cytokine–cytokine receptor interaction, viral protein interaction with cytokine and cytokine receptor, and HIF-1 signaling pathway), signal transduction (TNF signaling pathway, JAK-STAT signaling pathway, and PI3K-Akt signaling pathway), and immune system (chemokine signaling pathway, IL-17 signaling pathway, and Hematopoietic cell lineage). Notably, DEGs within these pathways were predominantly upregulated post-infection (PBS vs. Control), but downregulated following PG-1 or TET treatment (Table S5). Additionally, the PBS vs. control comparison shared 32 enriched pathways with PG-1 vs. PBS and 20 with TET vs. PBS comparison. Furthermore, the PG-1 vs. PBS comparison exhibited unique enrichment for 18 pathways compared to TET vs. PBS, with the most prominent being ECM–receptor interaction, osteoclast differentiation, and transcriptional misregulation in cancer. Finally, four pathways were uniquely enriched only in the PBS vs. control comparison, including pertussis, glycolysis/gluconeogenesis, biosynthesis of amino acids, and focal adhesion pathway (Figure 7). Within the enriched glycolysis/gluconeogenesis pathway (Table S5), 19 out of 26 (73.08%) DEGs were upregulated. The most significantly upregualted DEG was Phosphofructokinase (*Pfkl*), encoding the key rate-limiting enzyme for glycolysis, followed by Fructose-1,6-bisphosphatase 1 (*Fbp1*), which catalyzes the rate-limiting step in gluconeogenesis. In addition, genes encoding additional core glycolytic enzymes were significantly induced, including Hexokinase 2 (*HK2*), Hexokinase 3 (*HK3*), Phosphoglycerate Mutase 1 (*Pgam1*), Pyruvate Kinase M (*Pkm*), Phosphofructokinase (*Pfkp*), Lactate Dehydrogenase A (*Ldha*), Triosephosphate Isomerase 1 (*Tpi1*).

### 3.8. Validation of RNA-Seq Results by qRT-PCR

**Figure 8:**
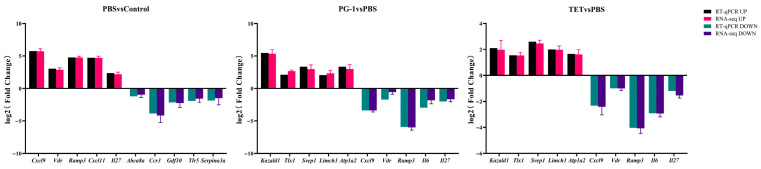
Experimental validations of selected 10 differentially expressed genes (DEGs) for each comparison group by the RT-qPCR method. Note: The bars in the histograms indicate the standard errors of mean expression levels in three replicates.

To validate the DEGs identified by RNA-seq, we performed qRT-PCR analysis on 10 randomly selected genes for each comparison group. As shown in Figure 8 (Table S6), the fold changes determined by qRT-PCR and RNA-seq were compared. All tested genes exhibited consistent expression trends between the two methods, supporting the reliability of the transcriptome data.

## 4. Discussion

The rise of multidrug-resistant *E. coli* has attracted substantial global concern in both human healthcare and veterinary medicine. Porcine ExPEC strains pose a particular threat due to their high prevalence and zoonotic potential [9,39]. Antimicrobial peptides (AMPs) have emerged as a promising therapeutic strategy. PG-1, a member of the AMP family, has been documented to exhibit broad-spectrum antimicrobial activities across multiple studies, possess immunomodulatory functions, and exert protective effects in an intestinal inflammation model, thereby suggesting its therapeutic utility in combating bacterial infections [15,16,23,26,40]. In this study, we systematically investigated the antibacterial efficacy, resistance induction potential, and immunomodulatory effects of PG-1 against the MDR porcine ExPEC strain PCN033, both in vitro and in a murine sepsis model.

### 4.1. PG-1 Demonstrates Potent and Resistance-Resistant Bactericidal Activity Against MDR Porcine ExPEC

Our previous research identified that the clinical porcine ExPEC strain PCN033 exhibits both multidrug resistance and virulence, harboring diverse antibiotic resistance genes on its chromosome and plasmids [10,27]. This study demonstrates that PG-1 exerts potent bactericidal activity against strain PCN033 with an MIC of 32 μg/mL, consistent with its reported activity spectrum against Gram-positive and Gram-negative bacteria [40,41]. The potential synergy between AMPs and conventional antibiotics represents a promising strategy to combat resistance development. Synergy, strictly defined by a FIC index of ≤0.5, indicates a combined effect greater than the sum of individual effects [42]. While such synergy has been documented, for instance, porcine myeloid AMP 36 with tetracycline/ gentamicin against PCN033 and PG-1 with colistin/gentamicin against Gram-negative *Acinetobacter baumannii* or gentamicin against Gram-positive *Micrococcus luteus* [43,44,45]. Our combination study revealed that PG-1 exhibited only an indifferent interaction (FIC = 1.03125) with tetracycline against PCN033. According to standard definitions [29], the observed indifferent effect indicates that the combination of PG-1 and tetracycline yielded no enhanced efficacy, with the overall activity attributable to the more potent agent alone. This outcome is mechanistically insightful: although the membrane-disrupting activity of PG-1 could theoretically promote the uptake of ribosomal-targeting antibiotics like tetracycline, the potent resistance mechanisms (e.g., efflux pumps) in PCN033 likely preclude any synergistic benefit. This finding highlights the context-dependency of synergy, which is governed by the specific antibiotic, bacterial strain, and its resistance arsenal. Consequently, the absence of synergy with tetracycline does not diminish the potential of PG-1 for collaborative action with other antibiotic classes. Future work should therefore prioritize systematic screening of PG-1 with membrane-impermeant antibiotics to identify partnerships capable of overcoming multidrug resistance. Notably, PG-1 maintained its MIC over 30 induction passages and 10 withdrawal generations, contrasting sharply with tetracycline’s progressive resistance development. The lack of induced resistance to PG-1 has also been observed in *Acinetobacter baumannii*, *Pseudomonas aeruginosa*, and methicillin-resistant *Staphylococcus aureus* [40,44]. These findings underscore potent antibacterial activity and exceptional antimicrobial stability of PG-1 against MDR ExPEC, highlighting its advantages over conventional antibiotics.

### 4.2. PG-1 Achieves Protective Efficacy with an Acceptable Safety Profile and Unique Immunomodulation

The clinical potential of PG-1 in vivo bacterial infection control relies on its protective efficacy but is constrained by hemolytic and cytotoxic effects. In a murine PCN033 infection model, PG-1 demonstrated significant protective effects while exhibiting 23.47 ± 1.91% hemolysis at therapeutic concentration, with cytotoxicity emerging at 2 × MIC (64 μg/mL) causing 51.82 ± 1.66% hemolysis, indicating acceptable biocompatibility within its therapeutic dosage range. Several promising approaches have been reported to optimize PG-1 by improving its selectivity, stability, and safety profile. These include modulating the charge and hydrophobicity of PG-1 via point mutation (e.g., V16R) to reduce hemolysis while retaining antimicrobial activity [46], employing targeted delivery approaches, such as fusion with an LPS-binding domain (Syn) to enhance serum stability and targeting [47], and improving biophysical properties like PEGylation promotes self-assembly into nanostructures to improve safety profiles [48]. However, the therapeutic efficacy of these optimized PG-1 analogs in clinical settings requires further evaluation. PG-1 significantly improved mouse survival compared to tetracycline post-PCN033 challenge. Both agents reduced bacterial loads in blood, brain, spleen, and lungs and mitigated histopathological damage, with PG-1 demonstrating superior efficacy. This protective effect aligns with validated efficacy of PG-1 in models of *P. aeruginosa*, *S. aureus*, and *Citrobacter rodentium* infection [26,40]. Furthermore, PG-1 transgenic mice conferred enhanced resistance to *Actinobacillus suis* infection and accelerated healing of *S. aureus*-infected wounds [49,50]. Critically, beyond direct antimicrobial properties of PG-1, its protective efficacy fundamentally involves immunomodulation. PCN033 infection triggered systemic inflammation, characterized by elevated pro-inflammatory cytokines (IL-1β, TNF-α) and suppressed anti-inflammatory cytokine TGF-β across in affected tissues, correlating with histopathologically confirmed pulmonary and cerebral lesions, consistent with the typical pathogenesis of ExPEC, which manifests as pneumonia, meningitis and septicemia [27,51]. PG-1 comprehensively modulated this response, suppressing IL-1β universally and TNF-α peripherally, while restoring TGF-β in circulatory and lymphoid tissues. In contrast, TET exerted limited immunoregulation, primarily within the spleen. This broad tissue-level cytokine reprogramming demonstrates unique capacity of PG-1 to reverse infection-driven immune dysregulation, surpassing conventional antibiotics.

### 4.3. PG-1 Coordinates Bactericidal Activity with Immunomodulation and Metabolic Intervention via Transcriptional Reprogramming

To elucidate anti-inflammatory mechanisms of PG-1, we pioneered comparative splenic transcriptomic analysis in PCN033-infected mice treated with PG-1 or TET. This analysis delineated infection-driven transcriptomic remodeling induced by PCN033 and PG-1-specific reprogramming of immune networks and metabolic pathways, contrasting with limited regulatory scope of TET. The most significantly upregulated gene upon PCN033 infection was *Cxcl9*, followed by *Cxcl11*. CXCL9, CXCL10, and CXCL11 are CXC chemokines that serve as the main ligands of C-X-C motif chemokine receptor 3 (*CXCR3*). The upregulation of *Cxcl9/10/11* promotes the clearance of intracellular pathogen *Mycobacterium tuberculosiscan* by recruiting Th1 cells and activating macrophages [52,53]. While *Cxcl9*, *Cxcl10* and *Cxcl11* were upregulated, *CXCR3* was downregulated post PCN033 infection. This apparent contradiction may reflect a precise immune feedback mechanism. During acute inflammation caused by PCN033 infection, *Cxcl9*, *Cxcl10* and *Cxcl11* induction recruits immune cells for PCN033 clearance. CXCR3 suppression limits excessive infiltration, preventing tissue damage, but calibrated immunosuppression may prolong pathogen resistance. Functional enrichment analysis revealed that PCN033 infection upregulated numerous immune pathways. GO analysis demonstrated enrichment of DEGs in biological processes, including immune system process and immune response, as well as in the molecular function category of cytokine activity. KEGG pathway analysis identified 10 significantly enriched immune system pathways, including Chemokine signaling, Toll-like/NOD-like receptor signaling, C-type lectin receptor signaling, Cytosolic DNA-sensing, IL-17 signaling, Th17 cell differentiation, Intestinal immune network for IgA production, Hematopoietic cell lineage, and complement and coagulation cascades. Further KEGG analysis revealed that PCN033 infection upregulated 20 genes in the NF-κB signaling pathway and 40 genes in the MAPK signaling pathway. This aligns with PCN033-induced upregulation of pro-inflammatory cytokines (IL-1β, IL-6, and IL-8) and activation of NF-κB/MAPK signaling pathway in porcine alveolar macrophage cell line 3D4/21 [54]. Consistent with this, our transcriptome data showed upregulated expression of *IL-1β* gene, corroborated by significantly elevated spleen IL-1β levels detected by ELISA. Additionally, PCN033 infection upregulated 19 genes encoding core enzymes for glycolysis and gluconeogenesis, and 30 of 38 genes enriched in the hypoxia-inducible factor 1 (HIF-1) signaling pathway, which is closely associated with glycolysis and critical for antibacterial immunity, suggesting metabolic reprogramming potentially supporting immune cell activation and pathogen adaptation. Under hypoxic conditions, HIF-1 levels quickly increase and enhance cellular glycolytic capacity by transcriptionally upregulating key glycolytic genes, including *HK2*, *Pfkfb*, *Pkm2*, and *Ldh*a [55,56,57], all of which were upregulated in murine spleens following PCN033 infection based on our transcriptomic data. These findings suggest that PCN033 infection may activate glycolysis in murine spleens through HIF-1 pathway, consistent with pathogenic bacterial glycolysis being key for host adaptation. Like other pathogenic *E. coli* using glycolysis for macrophage replication and intracellular pathogenesis during urinary tract infection [58,59], this activation of glycolysis by PCN033 may be a conserved strategy for it to adapt to the host environment and enhance its pathogenicity. We propose a model that PCN033-induced immune activation in the spleen drives high energy demand, met by accelerated glycolysis for ATP and metabolic intermediates, while elevated lactate fuels gluconeogenesis to sustain glucose supply, balancing energy flux and supporting immunity.

PG-1 treatment profoundly counteracted this infection signature. The most significantly downregulated gene was interleukin 6 (*Il6*). IL-6 is an important immunoregulatory and proinflammatory cytokine. IL-6 signaling plays a vital role in the control of the differentiation and activation of T lymphocytes [60]. Furthermore, PG-1 significantly downregulated key immune-associated genes, *Cxcl9*, *Cxcl11*, *Tnfrsf9*, *Vdr* and *Ramp3*, which were among the top 10 significantly upregulated genes post PCN033 infection. Tumor Necrosis Factor Receptor Superfamily Member 9 (Tnfrs9, also known as 4-1BB, CD137), an inducible T-cell costimulatory receptor, regulates diverse immune cell function and plays a dual role of immune enhancement and immune regulation [36]. Functional enrichment revealed a predominant downregulation trend in PG-1 vs. PBS comparison for DEGs enriched in critical immune pathways, including IL-17 signaling pathway, hematopoietic cell lineage, chemokine signaling pathway, Toll-like receptor (TLR) signaling pathway, TNF signaling pathway, JAK-STAT signaling pathway, MAPK signaling pathway, HIF-1 signaling pathway, and NF-κB signaling pathway, directly opposing the pan-upregulation induced by PCN033 infection. While this upregulation reflects essential host defense activation: The TLR pathway recognizes pathogen-associated molecular patterns (PAMPs) and activates the NF-κB/MAPK signaling axis, driving the release of proinflammatory cytokines [61,62,63,64]. The chemokine pathway recruits macrophages, neutrophils, and other immune cells to the spleen, while the hematopoietic cell lineage pathway differentiates bone marrow-derived immune cells, expanding the antibacterial cell reservoir [65]. IL-17 clears extracellular pathogens by recruiting neutrophils, and the TNF pathway enhances the phagocytic and bactericidal capacities of macrophages [66,67]. HIF-1 pathway maintains immune cell function under infection-induced hypoxia by promoting glycolytic energy supply [55]. While sustained hyperactivation of these pathways incurs splenic immunopathology. Persistent activation of NF-κB/TNF signaling causes cytokine cascade amplification, inducing immune cell apoptosis and edema [68,69]. IL-17-driven neutrophil infiltration releases reactive oxygen species and proteases that damage splenic architecture [70]. Abnormal upregulation of the hematopoietic cell lineage pathway may result in excessive proliferation of immune cells, disrupting immune homeostasis in the spleen. The downregulation of these pathways by PG-1 treatment reveals it dual anti-infective mechanisms: On one hand, PG-1 rapidly reduces pathogen load in the spleen by disrupting bacterial cell membranes and neutralizing lipopolysaccharide (LPS), decreasing upstream stimuli [17], leading to downregulation of the TLR/NF-κB signaling. Pathogen clearance also alleviate hypoxia in the infected microenvironment, which in turn reduces the demand for HIF-1 signaling, limits further immune cell recruitment, and downregulates chemokine and hematopoietic cell lineage pathway activity. On the other hand, PG-1 may actively temper overactivated immune pathways to prevent excessive immune responses. It inhibits bacterial phagocytosis by macrophages, key cells in initiating and amplifying innate immunity, thereby moderating harmful phagocytic overactivation. Furthermore, PG-1 exerts bactericidal effect with limited immune cell activation [16,17], demonstrating its ability to balance pathogen clearance while minimizing unnecessary immune overreaction. Supporting this anti-inflammatory profile, PG-1 downregulates the transcriptional expression of the innate immune gene *TLR2* in a murine model of *Citrobacter rodentium* intestinal infection [26]. Consequently, by modulating these pathways, PG-1 reduces proinflammatory cytokine release, limits excessive neutrophil infiltration, mitigates splenic tissue damage, and prevents self-inflicted damage caused by immune cell “overactivation.” Crucially, PG-1 achieves targeted attenuation, recalibrating pathway activity from pathological hyperactivation to homeostatic defense, preserving residual pathogen clearance while protecting splenic integrity. The regulation of the HIF-1 pathway provides a new perspective for understanding the “antimicrobial peptide-metabolism-immunity” crosstalk. These findings offer a theoretical basis for developing dual-function anti-infective drugs with both “bactericidal” and “immunomodulatory” properties, crucial for reducing post-infection immune organ damage. Study limitations include the need for protein-level validation of key pathway factors (e.g., phospho-NF-κB, STAT3 dimerization) to confirm functional pathway activity. Transcriptomic profiling also revealed that PG-1 treatment elicited substantially more DEGs than TET, with predominant enrichment in ECM–receptor interaction, osteoclast differentiation, and transcriptional misregulation in cancer pathways. This divergence reflects fundamental mechanistic distinctions. TET primarily exerts passive pathogen reduction Via ribosomal-targeted bactericidal mode, causing minimal host transcriptomic perturbation. While controlling infection, it fails to intervene in the immune imbalance or tissue damage, potentially delaying immune organ recovery. PG-1 demonstrates dual “antimicrobial-reparative” properties: potentially repairing infection-induced structural damage by regulating ECM–receptor interaction, balancing inflammation through cross-signaling nodes (such as NF-κB in the osteoclast differentiation pathway), and accelerating functional immune recovery by correcting transcriptional misregulation. PG-1 uniquely coordinates pathogen clearance with host homeostasis restoration, a capacity absent in conventional antibiotics like TET.

In summary, PG-1 represents a paradigm-shifting AMP that integrates potent, resistance-stable bactericidal activity against MDR ExPEC with multimodal immunomodulation. Its dual-action mechanism operates through direct pathogen clearance and precision tuning of immune–metabolic pathways to resolve inflammation and restore homeostasis. This bifunctional capacity positions PG-1 as a pioneering therapeutic blueprint for next-generation anti-infectives. To advance this potential, future work should focus on validating its efficacy across diverse clinical isolates and in physiologically relevant porcine models, as well as exploring synergy with immunomodulators to optimize clinical translation and address post-infectious complications.

## 5. Conclusions

This study was undertaken to comprehensively evaluate the therapeutic potential of PG-1 against MDR porcine ExPEC and to elucidate its underlying mechanisms. Our findings demonstrate that: (1) PG-1 exhibits potent and resistance-resistant bactericidal activity against the virulent strain PCN033 in vitro; (2) it confers significant protective efficacy in a murine systemic infection model with an acceptable safety profile; and (3) the protective effect is mechanistically underpinned by the unique capacity of PG-1 to reprogram hyperactivated immune and metabolic pathways, particularly NF-κB and HIF-1 signaling, thereby reversing infection-induced immunopathology. Collectively, this work positions PG-1 not merely as an antimicrobial agent but as a multifaceted therapeutic that simultaneously targets the pathogen and recalibrates the host response, offering a promising new strategy to combat challenging MDR bacterial infections.

## Figures and Tables

**Table 1:** qRT-PCR primer sequences.

Gene Name	Primer Sequence (5′-3′)	Product Length	GenBank Number
*GAPDH*	F: AATTCAACGGCACAGTCAAGG	137	14433
	R: ACCAGTAGACTCCACGACATAC		
*β-actin*	F: CCTGTATGCCTCTGGTCGTA	339	11461
	R: CGCTCGTTGCCAATAGTGAT		
*Kazald1*	F: CTGACAAATCCAGTTGTTGAGG	448	107250
	R: GTCCTTGCTACTACCATTATTG		
*Tlx1*	F: AGTGAGGTTAGCGTCCAG	138	21908
	R: CTCTCACCCTTCACTGTAAC		
*Svep1*	F: GTACTTCTGGCGGTGGAA	330	64817
	R: GAAGGTGCGGATTACAGAT		
*Limch1*	F: CGTGCCATAGAGGAAGTG	156	77569
	R: GATGATATGTCCGCACGAA		
*Atp1a2*	F: GCTCCAACTCTTCCTGAAT	101	98660
	R: ATATCTAGGTATCGTGCTAG		
*Cxcl9*	F: TGTTCTTGAAGGAGTCCAT	178	17329
	R: CTACACTGAAGAACGGAGAT		
*IL27*	F: CTTGAACGACGACGACTT	116	246779
	R: GGAAGAGGAGGAAGAAGAAG		
*Vdr*	F: AGTGAAGGAGCTGGTAGG	140	22337
	R: CATTGCCGAACACCTCTA		
*Ramp3*	F: GTCGGAGTTCATCGTGTATT	226	56089
	R: AGCCATAGCCACAGTCAG		
*Il6*	F: TAGTCCTTCCTACCCCAATTTCC	76	16193
	R: TTGGTCCTTAGCCACTCCTTC		
*Cxcl11*	F: TACTGCAATCCGGACCAGTG	122	56066
	R: GATGTTCGTGTGCCTCGTGA		
*Abca8a*	F: TATTGGCTTCTCCGAATGAA	152	217258
	R: GTGGTCCTGATGCTCCTT		
*Ccr3*	F: CTATGTTCTGTGGAATGAGTG	291	1277
	R: TCTGGATAGCGAGGACTG		
*Gdf10*	F: GGCTCGTGAGGTATTCTG	100	14560
	R: AAGACTGCGGAAGATTAGG		
*Tlr5*	F: CGAAGACTGCGATGAAGA	358	53791
	R: CTGGATGTTGGAGATATGGT		
*Serpina3a*	F: GTTCTCCTTATCACAAGACTAC	101	74069
	R: CTCCAGTGATCCCAGACA		

**Table 2:** Antibacterial activity of PG-1 and TET against PCN033 and *E. coli* ATCC25922.

Antimicrobial Agents	PCN033		*E. coli* ATCC25922	
	MIC (μg/mL)	MBC (μg/mL)	MIC (μg/mL)	MBC (μg/mL)
PG-1	32	32	32	64
TET	512	512	0.125	0.25

**Table 3:** Minimum Inhibitory Concentrations (MICs) of PG-1 and TET alone and in combination, and their Fractional Inhibitory Concentration (FIC) Indices.

Antimicrobial Agents	MIC (μg/mL)				FIC
	PG-1		TET		
	Single Drug	Combination Therapy	Single Drug	Combination Therapy	
*E. coli* ATCC25922	32	32	0.25	0.03125	1.125
PCN033	32	32	512	16	1.03125

**Table 4:** Summary statistics of the spleen RNA-Seq data.

Sample	Raw Reads	Clean Reads	Clean Bases	Q30 (%)	GC (%)	Total Mapped Reads
PBS1	46,004,704	44,834,110 (97.46%)	6.73 G	97.28	49.74	39,676,052 (88.5%)
PBS2	49,219,132	48,065,826 (97.66%)	7.21 G	97.19	49.93	42,553,506 (88.53%)
PBS3	44,484,664	43,692,736 (98.22%)	6.55 G	97.38	50.3	38,977,111 (89.21%)
PG1-1	40,395,778	39,509,588 (97.81%)	5.93 G	97.41	48.9	35,492,724 (89.83%)
PG1-2	48,340,560	47,100,692 (97.44%)	7.07 G	97.45	49.41	42,595,633 (90.44%)
PG1-3	52,695,198	50,631,020 (96.08%)	7.59 G	97.3	49.8	45,094,025 (89.06%)
TET1	50,118,192	48,757,348 (97.28%)	7.31 G	97.33	49.74	42,893,924 (87.97%)
TET2	41,649,796	40,517,388 (97.28%)	6.08 G	97.25	50.09	35,941,515 (88.71%)
TET3	47,035,952	45,557,334 (96.86%)	6.83 G	97.31	49.75	40,320,159 (88.5%)

## Data Availability

The data presented in this study are openly available in the NCBI Sequence Read Archive at Bioproject, reference number PRJNA1280477.

## Supplementary Materials

The following supporting information can be downloaded at: https://www.mdpi.com/article/10.3390/vetsci12111030/s1, Table S1: Comprehensive Evaluation of Antibacterial Activity, Biosafety, and Therapeutic Efficacy; Table S2: Differentially Expressed Genes (DEGs); Table S3: The top ten up/down-regulated DEGs; Table S4: GO Enrichment; Table S5: KEGG Enrichment; Table S6: Differential Gene Expression Profile (Log2Fold Change); Figure S1: KEGG pathway analysis of DEGs in spleen tissue under different comparison groups. Figure S2: Experimental Flow Diagram.

## Abbreviations

The following abbreviations are used in this manuscript:

ExPEC	extraintestinal pathogenic *Escherichia coli*
PG-1	Protegrin-1
MIC	minimal inhibitory concentration
AMP	Antimicrobial peptide
MDR	multidrug-resistant
MBC	Minimum bactericidal concentration
FIC	fractional inhibitory concentrations
TET	tetracycline
DEGs	Differentially expressed genes
GO	Gene Ontology
KEGG	Kyoto Encyclopedia of Genes and Genomes
PEG	polyethylene glycol
