# Supplementary material for: Protegrin-1 Combats Multidrug-Resistant Porcine ExPEC: Potent Bactericidal Activity and Multimodal Immunometabolic Regulation In Vitro and in a Murine Model
Source: Vet Sci. 2025 Oct 23;12(11):1030. doi: 10.3390/vetsci12111030 (PMC12656810; doi:10.3390/vetsci12111030)
Supplement: Supplementary file 1 [file vetsci-12-01030-s001.zip › Figure S2. Experimental Flow Diagram.pdf]

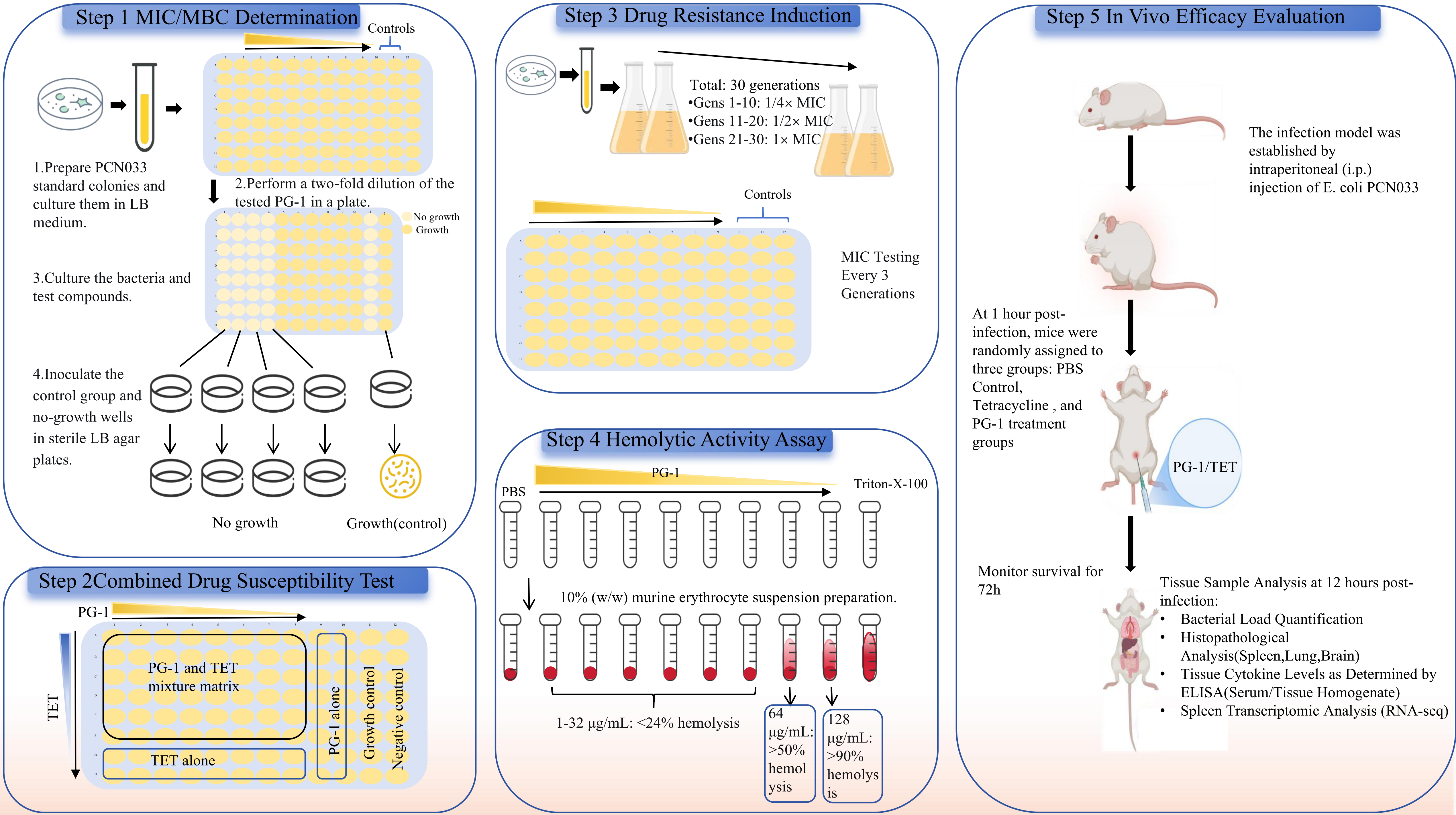
Step 1 MIC/MBC Determination
Controls
1.Prepare PCN033 standard colonies and culture them in LB medium.
2.Perform a two-fold dilution of the tested PG-1 in a plate.
No growth
Growth
3.Culture the bacteria and test compounds.
4.Inoculate the control group and no-growth wells in sterile LB agar plates.
No growth
Growth(control)
Step 2Combined Drug Susceptibility Test
PG-1
TET
PG-1 and TET mixture matrix
TET alone
PG-1 alone
Growth control
Negative control
Step 3 Drug Resistance Induction
Total: 30 generations
•Gens 1-10: 1/4× MIC
•Gens 11-20: 1/2× MIC
•Gens 21-30: 1× MIC
Controls
MIC Testing Every 3 Generations
Step 4 Hemolytic Activity Assay
PG-1
PBS
Triton-X-100
10% (w/w) murine erythrocyte suspension preparation.
1-32 μg/mL: <24% hemolysis
64 μg/mL: >50% hemolysis
128 μg/mL: >90% hemolysis
Step 5 In Vivo Efficacy Evaluation
The infection model was established by intraperitoneal (i.p.) injection of E. coli PCN033
At 1 hour post-infection, mice were randomly assigned to three groups: PBS Control, Tetracycline , and PG-1 treatment groups
PG-1/TET
Monitor survival for 72h
Tissue Sample Analysis at 12 hours post-infection:
• Bacterial Load Quantification
• Histopathological Analysis(Spleen,Lung,Brain)
• Tissue Cytokine Levels as Determined by ELISA(Serum/Tissue Homogenate)
• Spleen Transcriptomic Analysis (RNA-seq)
