# Supplementary figures and images for: Protegrin-1 Combats Multidrug-Resistant Porcine ExPEC: Potent Bactericidal Activity and Multimodal Immunometabolic Regulation In Vitro and in a Murine Model
Source: Vet Sci. 2025 Oct 23;12(11):1030. doi: 10.3390/vetsci12111030 (PMC12656810; doi:10.3390/vetsci12111030)

# KEGG pathway annotation

## PBS vs Control

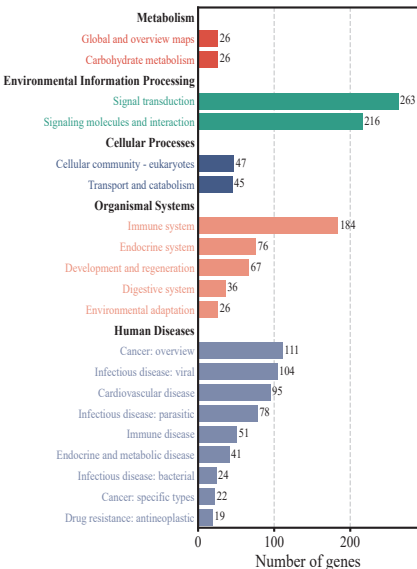

## PG-1 vs PBS

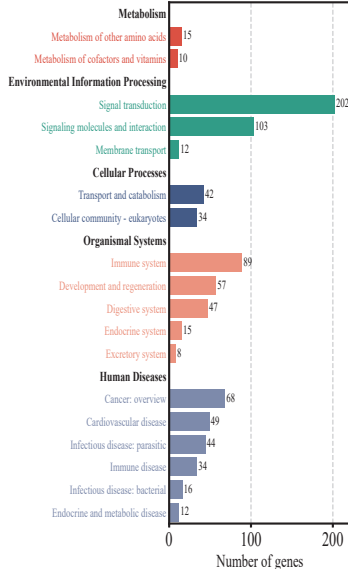

## TET vs PBS

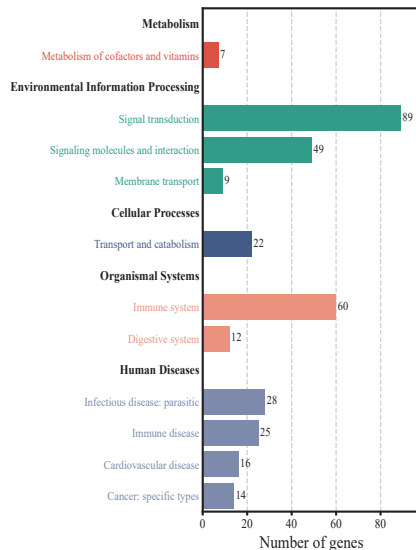

Supplement: Supplementary file 1 [file vetsci-12-01030-s001.zip › Figure S1. KEGG pathway analysis of DEGs in spleen tissue under different comparison groups.pdf]
